# A gastrointestinal rotavirus infection mouse model for immune modulation studies

**DOI:** 10.1186/1743-422X-8-109

**Published:** 2011-03-08

**Authors:** Karen Knipping, Monica M McNeal, Annelies Crienen, Geert van Amerongen, Johan Garssen, Belinda van't Land

**Affiliations:** 1 Danone Research Centre for Specialised Nutrition, P.O. Box 7005, 6700 CA Wageningen, The Netherlands; 2 Cincinnati Children's Hospital Medical Center, Division of Infectious Diseases, 3333 Burnet Ave, Cincinnati, OH 45229 3039, USA; 3 Former employee of Danone Research Centre for Specialised Nutrition, P.O. Box 7005, 6700 CA Wageningen, The Netherlands; 4 Dutch Vaccine Institute, P.O. Box 457, 3720 AL Bilthoven, The Netherlands; 5 Utrecht Institute for Pharmaceutical Sciences, P.O. Box 80115, 3508 TC Utrecht, The Netherlands

## Abstract

**Background:**

Rotaviruses are the single most important cause of severe diarrhea in young children worldwide. The current study was conducted to assess whether colostrum containing rotavirus-specific antibodies (Gastrogard-R^®^) could protect against rotavirus infection. In addition, this illness model was used to study modulatory effects of intervention on several immune parameters after re-infection.

**Methods:**

BALB/c mice were treated by gavage once daily with Gastrogard-R^® ^from the age of 4 to 10 days, and were inoculated with rhesus rotavirus (RRV) at 7 days of age. A secondary inoculation with epizootic-diarrhea infant-mouse (EDIM) virus was administered at 17 days of age. Disease symptoms were scored daily and viral shedding was measured in fecal samples during the post-inoculation periods. Rotavirus-specific IgM, IgG and IgG subclasses in serum, T cell proliferation and rotavirus-specific delayed-type hypersensitivity (DTH) responses were also measured.

**Results:**

Primary inoculation with RRV induced a mild but consistent level of diarrhea during 3-4 days post-inoculation. All mice receiving Gastrogard-R^® ^were 100% protected against rotavirus-induced diarrhea. Mice receiving both RRV and EDIM inoculation had a lower faecal-viral load following EDIM inoculation then mice receiving EDIM alone or Gastrogard-R^®^. Mice receiving Gastrogard-R^® ^however displayed an enhanced rotavirus-specific T-cell proliferation whereas rotavirus-specific antibody subtypes were not affected.

**Conclusions:**

Preventing RRV-induced diarrhea by Gastrogard-R^® ^early in life showed a diminished protection against EDIM re-infection, but a rotavirus-specific immune response was developed including both B cell and T cell responses. In general, this intervention model can be used for studying clinical symptoms as well as the immune responses required for protection against viral re-infection.

## Background

Rotavirus is one of the leading causes of severe dehydrating diarrhea in children under the age of five and causes the deaths of >600,000 children annually [1]. Rotaviruses, belonging to a genus of double-stranded RNA viruses in the family Reoviridae, infect the mature villus epithelial cells of the small intestine, often leading to fever, vomiting, and diarrhea in children. Current treatment is non-specific and consists mainly of oral rehydration therapy to prevent dehydration. Two live-attenuated vaccines have been licensed recently and have so far proven safe and efficacious [1]. However, previous experience with the first licensed rotavirus vaccine, which was withdrawn from the market a year after introduction due to a possible correlation between vaccine application and the occurrence of intussusceptions [2], has reinforced the need to develop alternative approaches to control rotavirus disease. Fundamental to this development is a better insight of the immune responses related to gastrointestinal virus infections which will help to develop improved treatment and/or preventive regimes.

Mice provide a reliable animal model for studying the immune responses during a primary rotavirus infection, although the kinetics of rotavirus infections in mice differs slightly from what is observed in humans [3]. Unlike infant mice which are susceptible to symptomatic infection with rotavirus only during the first 15 days of life, human infants can suffer from multiple rotavirus infections up to the age of five years. There are even many reports of adult rotavirus infection, particularly in the elderly [4]. Aside from these differences, studies of rotavirus infection in mice can provide valuable information on the induction of immune responses by the virus. Sheridan et al. was one of the first to describe a mouse model studying rotavirus-specific immunity. Their findings indicate that (i) infection occurs in all age groups but diarrheal disease is observed in neonatal animals only and that (ii) re-infection of adult animals is associated with suppression of virus-specific cell-mediated immunity [5].

Despite many years of research, the immune correlates of protection from rotavirus infection and disease are still not completely understood. The mouse model has been extensively used to investigate the contribution of different components of the immune system necessary for protection. These studies have suggested that both humoral- and cell-mediated immunity are important in the resolution of ongoing rotavirus infection and in protection against subsequent re-infection [6]. In more detail, studies have shown that B cells were essential for long-term protection against rotavirus [7]. CD4^+ ^T cells were pivotal for the development of approximately 90% of the rotavirus-specific intestinal IgA. Their presence seems to be critical for the establishment of protective long-term memory responses and IgA antibody in serum and stool samples correlates best with protection against re-infection [8,9]. CD8^+ ^T cells appeared to be involved in providing partial protection against re-infection [10,11].

The original neonatal mouse model has been developed in order to determine the effects of an immature immune system on responses to candidate vaccines [12]. In the present study, this model has been modified to a sensitive gastrointestinal viral infection and illness model in infant mice for testing nutritional compounds for their antiviral and/or immunomodulatory activity. Therefore, neonatal mice were inoculated with a rhesus rotavirus strain (RRV) at an early age for immune induction and later challenged with epizootic-diarrhea infant-mouse (EDIM) virus. Protection due to intervention with the nutritional supplement Gastrogard-R^® ^was determined by reduction of diarrhea and protection against later EDIM challenge, measured by rotavirus fecal-shedding. Associations between protection and both humoral (antibody) and cellular (T cell) responses were examined.

Gastrogard-R^® ^is prepared from the colostrum of hyperimmunised cows and contains high antibody titers against four human rotavirus serotypes, as measured in a virus neutralisation test [13]. It is used as prophylactic treatment of 'at risk' children aged one month to three years to prevent diarrhea due to rotavirus infection and the efficacy of treatment was established in a clinical trial in children aged 3 to 15 months [13].

The purpose of this study was to demonstrate a modified gastro-intestinal viral re-infection model for studying the effects of nutritional intervention on clinical symptoms as well as the development of immune responses and protection against subsequent viral infection.

## Methods

### Viruses

To obtain a large quantity of virulent epizootic-diarrhea infant-mouse virus (EDIM), 78 neonatal mice were inoculated at the age of 4 days with 5 μl EDIM 7.8*10^7 ^focus forming units/ml, a kind gift from Dr. Richard Ward, Cincinnati Children's Hospital Medical Center, USA. Stool samples were collected and pooled from day 5 until day 13 and EDIM was extracted with genetron (1,1,2-trichloro-1,2,2,-trifluoroethane, Sigma-Aldrich, Zwijndrecht, The Netherlands). The stool preparation of EDIM contained 400 μg rotavirus/ml as determined in the IDEIA ELISA (Dako diagnostics, Glostrup, Denmark) verified against a rotavirus stock with known concentration of rotavirus. To determine whether EDIM caused diarrheal disease when orally administered to neonatal mice, 7 mice were inoculated with 5 μl EDIM (400 μg rotavirus/ml) at the age of 7 days. At day 2 p.i., 70% of the mice were suffering from diarrhea, rising to 100% at day 5 and declining again from day 6.

The rhesus rotavirus (RRV) strain used in this study, also provided by Dr. Richard Ward, was grown in African green monkey kidney MA104 cells (ECACC, Salisbury, UK) and concentrated by ultracentrifugation. The titer was determined using a titration assay in MA104 cells resulting in a 50% cell culture infective dose (CCID_50_) of 1 × 10^7.7^. Virus stocks were UV-inactivated overnight at short wave UV 254 nm (UV cabinet CM-10; Alltech, Breda, The Netherlands) and used for measuring delayed-type hypersensitivity (DTH) responses and T cell stimulation. Inactivation of rotavirus was confirmed using the titration assay in MA104 cells. For DTH responses, the EDIM and RRV stocks were diluted 100× in PBS. For use in T cell stimulation experiments, caesium chloride gradient purified EDIM double layered particles (provided by Dr. Richard Ward) were diluted to 1 μg/ml and RRV was diluted to a CCID_50 _of 5 × 10^7^.

Simian rotavirus, SA-11, (ATCC, Middlesex, UK) was grown in MA104 cells and concentrated by ultracentrifugation. SA-11 contained 14 μg rotavirus/ml as determined in the IDEIA ELISA, verified against a rotavirus stock with known concentration of rotavirus. For the determination of rotavirus-specific antibodies in serum a concentration of 500 ng/ml SA-11 was used.

### Mice

Pregnant female BALB/c mice were housed individually in sterile micro-isolation cages under standard housing conditions at the animal facility of the Dutch Vaccine Institute with a 12 h dark and light cycle. Animal care and use were performed in accordance with the guidelines of the Dutch Committee of Animal Experiments. Weaning of suckling pups occurred at 21 days of age. Alternatively, pups immunized and challenged prior to weaning remained with their dams.

### Study design

**Figure 1:**
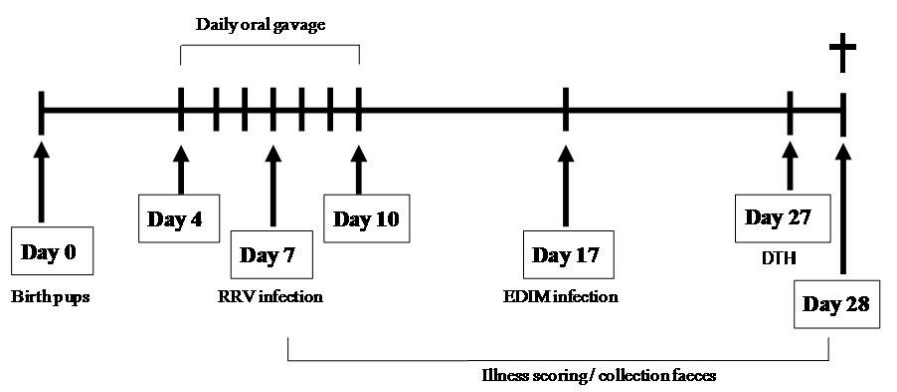
**Schematic design of the rotavirus double infection model**. At day 0 the pups were born. From day 4 until day 10 after birth pups one group received intervention of rotavirus-specific antibodies (Gastrogard-R^®^) by oral gavage. At day 7 of age pups were inoculated with RRV by oral gavage. From day 8 on, the number of pups experiencing illness and the severity of illness was scored daily. From day 8 until the end of the experiment, feces were collected. At day 16 a blood sample was collected and at day 17 of age pups were inoculated with EDIM by oral gavage. At day 27 of age a rotavirus-specific DTH was elicited by subcutaneous inoculation of rotavirus in the ear pinnea, EDIM in the left ear and RRV in the right ear. DTH responses were measured 24 hours later, prior to collection of spleen and blood samples.

A total of 91 neonatal mice were cross-fostered and divided into 4 study groups as depicted in Table 1. A schematic design of the mouse model is shown in Figure 1. From day 4 until day 10 of age, pups of group D received daily 20 μl PBS containing rotavirus-specific antibodies, Gastrogard-R^®^, (kindly provided by Dr. Rosie Pereira) (1 mg/day) by oral gavage. At day 7 of age, pups of group A, C and D were inoculated by oral gavage with 10 μl RRV (CCID_50 _1 × 10^7.7^) and mice of group B were inoculated with 10 μl PBS as a control. From day 8 until day 14, diarrhea and severity of illness was scored. From day 8 until day 27 feces were collected daily, the feces of all animals within one group were pooled each day. At day 16 of age, a blood sample was taken and pooled from all animals within one group. At day 17 of age, pups of group B, C and D were inoculated with 5 μl EDIM (400 μg rotavirus/ml) and mice of group A were inoculated with 10 μl PBS as a control. At day 27 of age a rotavirus-specific DTH was elicited by subcutaneous inoculation of UV-inactivated rotavirus in the ear pinnea, EDIM (1 μg/ml) in the left ear and RRV (CCID_50 _5 × 10^7^) in the right ear. Non-inoculated mice of comparable age were also injected as a control. Twenty four hours later, the DTH responses were determined by measuring ear thickness using a digital micrometer (Mitutoyo, Veenendaal, The Netherlands) prior to collection of individual spleen and individual blood samples.

**Table 1:** Study design of inoculation of mice with RRV and/or EDIM and intervention.

Group	n (pups)	RRV at day 7 of age	EDIM at day 17 of age	Intervention at day 4 - 10 of age
Group A	22	x	-	-
Group B	23	-	x	-
Group C	24	x	x	-
Group D	22	x	x	Gastrogard-R^®^

Group A received RRV only, group B received EDIM only as controls. Groups C and D received both RRV and EDIM. Group D received daily 20 μl PBS containing rotavirus-specific antibodies (Gastrogard-R^®^) 1 mg/day.

### Diarrhea and severity of illness scoring

Daily evaluation, starting 1 day after RRV inoculation, for the presence of diarrhea (defined as having diarrheal stool after gentle palpation of the abdomen) was performed for each animal, and results were reported as the percent of animals having diarrhea in each group. Stool samples were collected daily, the feces of all animals within one group were pooled each day. The severity of illness was scored daily by assigning numeric values to the color of stool where a high score indicates severe illness (yellow = 3; yellow-brown = 2; brown = 1), degree of soiling (very soiled = 4; somewhat soiled = 1; no soiling = 0), and consistency (very liquid = 4; liquid = 3; solid = 1) of the stool. The severity score was calculated by dividing the total severity score by the total number of animals on each day after RRV immunization [12].

### Detection of rotavirus in feces

A commercial ELISA kit (IDEIA; Dako diagnostics) for the detection of group A rotavirus in human fecal samples was used according to the manufacturer's protocol. In short, precoated wells with rotavirus-specific polyclonal antibody were sampled with a reference EDIM stock with known concentration (300 ng/ml) which was used as a standard, stool samples (dilutions 5×, 10×, 50× and 100×), positive and negative control supplied in the kit. Then 100 μl rotavirus-specific polyclonal antibody-peroxidase labeled was added and incubated for 1 hour at RT. Wells were washed with 350 μl wash buffer and incubated with 100 μl of TMB substrate for 10 min. at room temperature (RT). The reaction was stopped by adding 100 μl of 0.46 mol/L sulfuric acid and the absorbance was measured at 450 nm on a microplate reader (BioRad, Hemel Hempstead, UK). The ELISA cutoff was the mean absorbance of the negative control at 450 nm plus a factor of 0.1. The absorbance of the positive control should be in the range indicated at the validation inlay in the kit. Concentration of rotavirus in the samples was expressed in ng/ml.

### *In vitro *Concanavalin A and rotavirus-specific proliferation of spleen cells

Spleen cells of individual mice of group A (n = 22), group B (n = 23), group C (= 24), group D (n = 22) and a control group of non-inoculated mice (n = 15) were isolated by using a cell strainer 40 μm (BD Falcon, Breda, The Netherlands) to a single-cell suspension. The cells were incubated with 5 ml ice-cold lysis buffer (4.15 g ammonium chloride (Merck, Haarlem, The Netherlands) + 0.5 g potassium bicarbonate (Sigma-Aldrich, Zwijdrecht, The Netherlands) + 18.6 mg EDTA (Sigma-Aldrich) in 500 ml water, pH 7.3) and incubated for 5 minutes on ice for lysing red blood cells. After incubation, 10 ml of ice-cold culture medium, RPMI-1640 (Life Technologies, Breda, The Netherlands) + heat inactivated 10% FBS (Perbio Science, Etten-Leur, The Netherlands) + penicillin 50 U/ml and streptomycin 50 μg/ml (Life Technologies) + 1% sodium pyruvate (Life Technologies) was added. The cell suspension was centrifuged for 5 min. at 400 × g and 4°C (Sorvall RT7, Thermo Fisher Scientific, Breda, The Netherlands) and resuspended in 2 ml ice-cold culture medium. For the Con A type IV (Sigma-Aldrich) stimulation, 2 × 10^5 ^cells were stimulated with 3 μg/ml Con A. For the rotavirus-specific stimulation, 1 × 10^6 ^cells were stimulated with 5 × 10^7 ^CCID_50 _UV-inactivated RRV or 1 μg/ml UV-inactivated EDIM dl particles. Plates were incubated at 37°C and 5% CO_2_. Con A proliferated cells were pulsed after 24 hours and the rotavirus-specific proliferated cells after 5 days with 0.4 μCi/well tritium-thymidine (PerkinElmer, Groningen, The Netherlands) and incubated overnight. Cells were harvested on filter plates (Unifilter GF-C; PerkinElmer) and radioactivity was determined in 25 μl of scintillation cocktail (Ultima gold; PerkinElmer) in a Wallac MicroBeta liquid scintillation detector (PerkinElmer). Stimulation index was calculated as the ratio of counts per minute for antigen-stimulated cultures to background cultures.

### Detection of serum rotavirus-specific IgM, IgG and IgG-subclass antibodies

Serum samples were collected from mice bled from the orbital sinus and centrifuged 10 minutes at 400 × g (Sorvall RT7; Thermo Fisher Scientific). Wells of a 96-well plate (BD Falcon, Breda, The Netherlands) were coated overnight with 100 μl 500 ng/well of simian rotavirus (SA-11) at 4°C. As described previously, SA-11 is an efficient EDIM antigen substitute in an ELISA [5]. Wells were washed 4× with 200 μl PBS (Life Technologies) + 0.05% Tween 20 (Merck) and blocked for 30 min. at 37°C with 200 μl assay buffer (PBS + 0.5% BSA (MP Biomedicals, Eindhoven, The Netherlands) + 0.05% Tween 20). As reference serum, the sera from pups and mothers from a previous EDIM passage experiment that was shown to contain antibodies to rotavirus were used. For each isotype or subclass, a different reference serum with the highest titer, tested in a serial dilution series starting from a 1:10 dilution, was selected and set to the arbitrary unit (AU) of 100. At day 16, individual serum samples were collected and then pooled per group. At day 28 individual serum samples were collected and were tested individually. Serial dilutions in 100 μl were made of the reference serum and individual serum samples, starting from a 1:10 dilution in assay buffer. Wells were washed as described above and incubated for 1 hour at 37°C with 100 μl 1:7,500 goat anti-mouse IgM μ-chain-HRP (Sigma-Aldrich) or 1:1,000 goat anti-mouse IgG-HRP (Tebu-Bio, Heerhugowaard, The Netherlands) or 1:5,000 goat anti-mouse IgG1-HRP (AbD Serotec, Düsseldorf, Germany) or 1:1,000 goat anti-mouse IgG2a-HRP (AbD Serotec) or 1:1,000 goat anti-mouse IgG2b-HRP (AbD Serotec) or 1:1,000 goat anti-mouse IgG3-HRP (AbD Serotec) in assay buffer. Plates were washed, 100 μl 3,3',5,5'-tetramethylbenzidine (TMB; Perbio Science) was added and incubated for 10 min at room temperature. The reaction was stopped with 100 μl 10% sulphuric acid (Merck) and the absorbance measured at 450 nm on a microplate reader (BioRad). Results were calculated against the reference serum and expressed in AU. Limit of detection: 0.15 AU for IgG and IgM, 2.5 AU for IgG1, 0.6 AU for IgG2a, 0.15 AU for IgG2b and 0.3 AU for IgG3.

### Statistical analysis

Power calculation indicated a group size of at least 13 animals to find significant differences in diarrheal score (detectable difference in diarrheal score = 20%, expected CV = 15%, α = 0.05, β = 0.20). All statistical analyses were performed using the statistical software package GraphPad Prism, version 4.03. A two-sample t test was used when two groups were compared. Values of P less than 0.05 were considered significant.

## Results

### Diarrhea and severity of illness score of RRV infected pups

**Figure 2:**
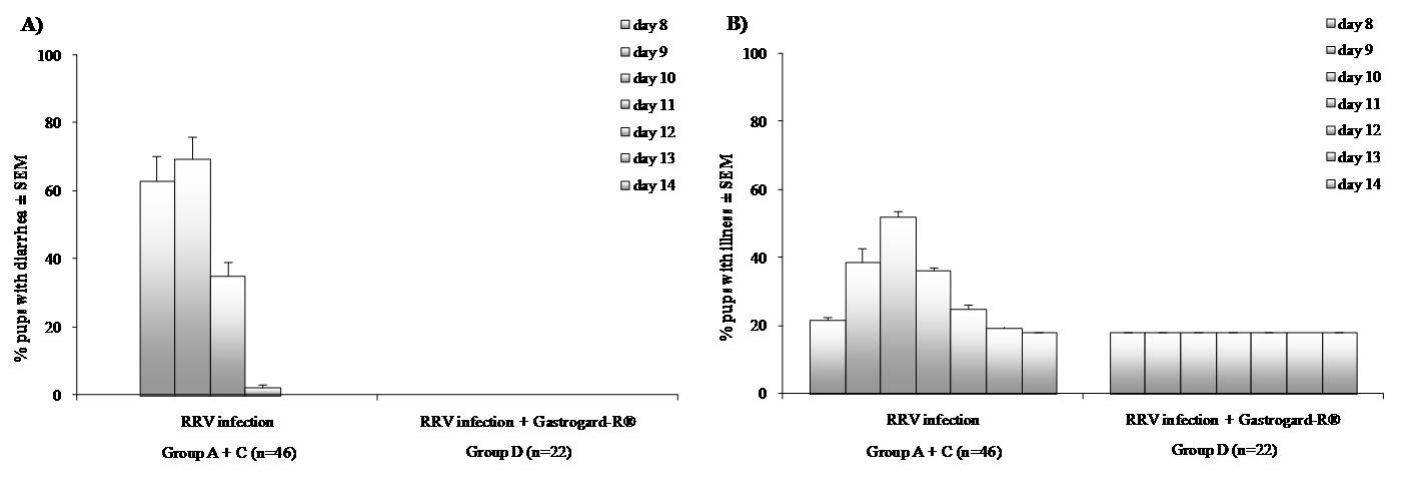
**Diarrhea and illness severity score after RRV infection**. Percent of pups suffering from diarrhea (A) during RRV infection of group A and C combined (n = 46) administered at day 7 and of pups receiving rotavirus-specific antibodies (RRV infection + Gastrogard-R^®^; n = 22)). Diarrhea was monitored from day 8 until day 14. Bars represent the mean percentage of pups ± SEM per day having symptoms of diarrhea. Severity of illness score (B) during RRV infection of group A and C combined (n = 46) administered at day 7 and of pups receiving rotavirus-specific antibodies (RRV infection + Gastrogard-R^®^; n = 22). Severity of illness was monitored daily by assigning numeric values to the color of stool where a high score indicates severe illness (yellow = 3; yellow-brown = 2; brown = 1), degree of soiling (very soiled = 4; somewhat soiled = 1; no soiling = 0), and consistency (very liquid = 4; liquid = 3; solid = 1) of the stool. Bars represent the mean percentage of severity of illness score of the pups ± SEM per day.

Diarrhea and severity of illness was monitored daily during the first RRV infection from day 8 (1 day p.i.) until day 14 of age. Diarrhea in mice inoculated with RRV (group A and C combined) appeared at day 9 with 63% of the pups having symptoms of diarrhea, rising to a maximum of 70% at day 10 and declining to zero at day 13 (Figure 2A). When the mice were supplemented with rotavirus-specific antibodies (Gastrogard-R^®^) orally (group D) prior and during the RRV infection, no signs of diarrhea occurred indicating a complete protection against RRV induced diarrhea.

The severity of illness score, as depicted in Figure 2B, was monitored daily by assigning numeric values to the color of stool. A high score indicates severe illness, minimum score is 2 (18.2%) and maximum score is 11 (100%). Illness in mice inoculated with RRV (group A and C combined) appeared at day 9 with an average severity score of 38.6%, reaching a maximum of 52.3% at day 10 and declining after day 11. Mice supplemented with Gastrogard-R^® ^orally prior and during the RRV infection showed no signs of illness.

### Viral shedding of RRV in feces

Viral shedding in the feces was detected by measuring the amount of virus antigen shed after the RRV inoculation at day 7. Rotavirus in the feces after RRV inoculation was detectable but in low levels (between 15-130 ng/ml) during RRV infection (group A and C), but no rotavirus was detected in the Gastrogard-R^® ^group (group D) (data not shown).

### Viral shedding of EDIM in feces

**Figure 3:**
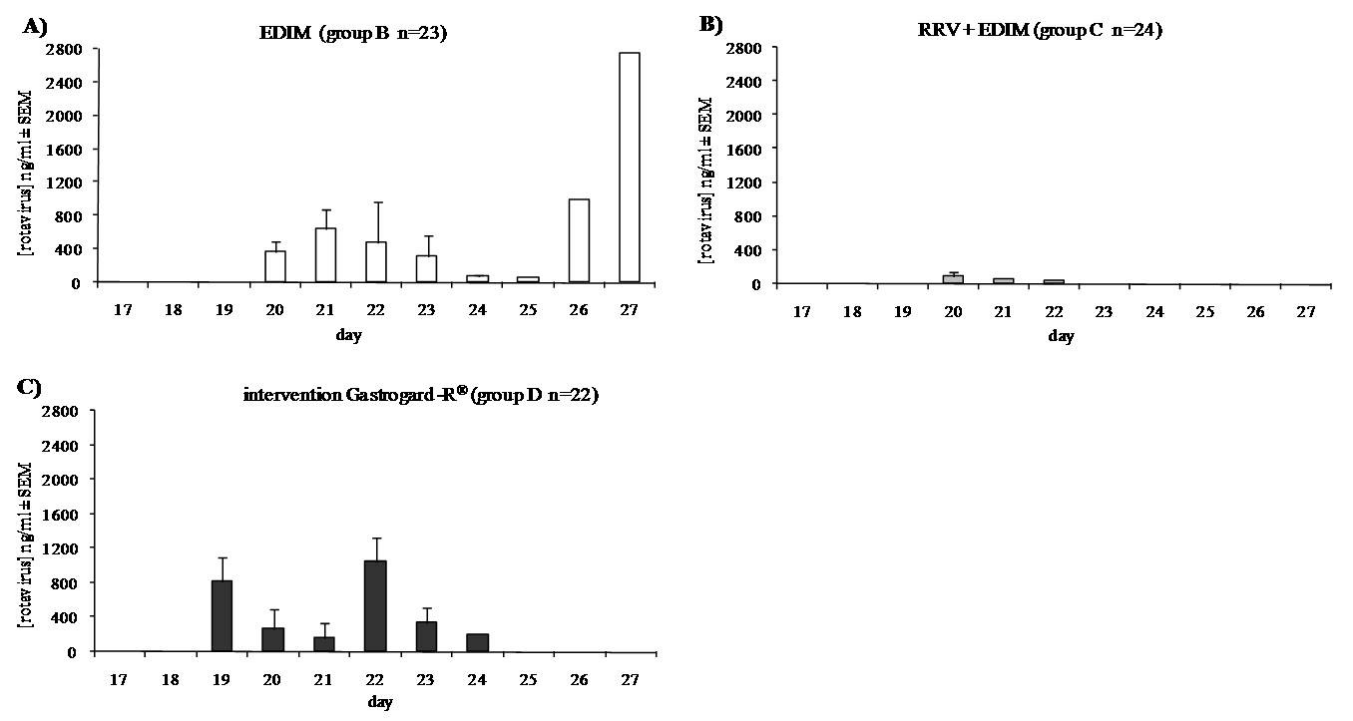
**Viral shedding in feces after EDIM infection**. Detection of viral shedding by measuring the concentration of rotavirus (ng/ml) ± SEM in feces after the secondary EDIM inoculation at day 17 of age. Group B was inoculated with only EDIM (A), group C was inoculated with RRV and EDIM (B) and group D was inoculated with both RRV and EDIM and received rotavirus-specific antibodies (Gastrogard-R^®^) prior and during RRV infection (C).

Viral shedding in the feces was detected by measuring the amount of virus antigen shed after the EDIM inoculation at day 17. The results of the viral shedding after the EDIM inoculation are depicted in Figure 3. A primary infection with RRV decreased viral shedding by 81% during a secondary infection with EDIM (Figure 3B) compared to infection with EDIM alone (Figure 3A). Similar findings have previously been described with heterologous rotavirus infection in mice [8,12]. Intervention with Gastrogard-R^® ^had 100% protected the mice against RRV induced diarrhea, but viral shedding during the secondary infection with EDIM showed no significant difference compared to the EDIM alone group (Figure 3C). However, in the EDIM group, shedding of rotavirus was measured up until day 27, whereas in the Gastrogard-R^® ^group, no rotavirus was detected in the feces after day 24. This might indicate that passive protection against a primary illness does not protect against viral shedding during a secondary infection, though clearing of rotavirus seemed more rapid in the Gastrogard-R^® ^group than in mice inoculated with EDIM alone.

### Delayed-type hypersensitivity (DTH) reaction to rotavirus EDIM and RRV

**Figure 4:**
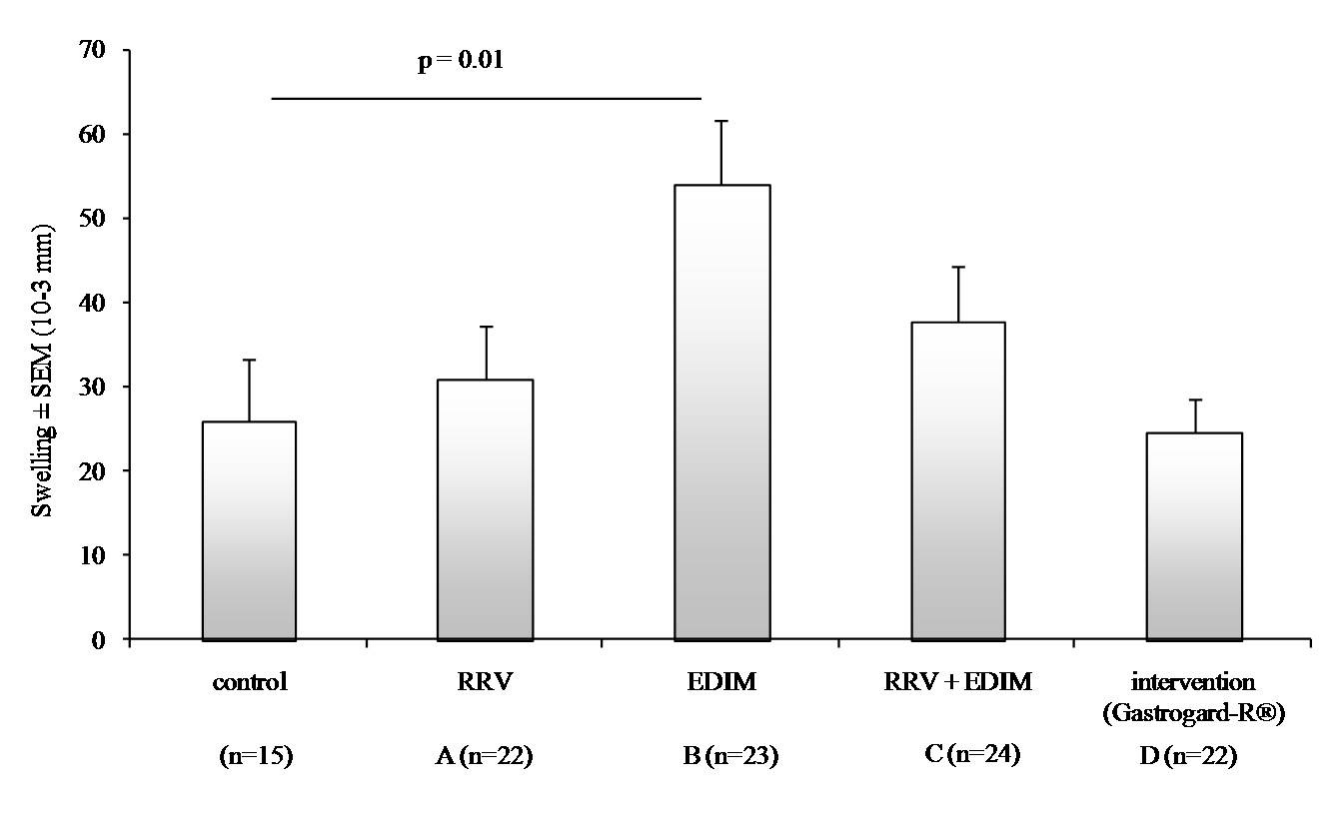
**DTH responses to rotavirus**. DTH response to EDIM by measuring ear thickness 24 hours after administration of EDIM in the left ear pinnea. Bars represent the mean ear swelling (10^-3 ^mm) per group ± SEM. EDIM induced a significant (p = 0.01) increase in ear swelling in the EDIM group compared to the control. The RRV, RRV + EDIM and Gastrogard-R^® ^groups were not significantly different from the control.

To analyze RRV- and EDIM-specific cell-mediated immunity of all study groups, a DTH response was measured by subcutaneous injection of rotavirus in the ear pinnea, RRV CCID_50 _of 2.5 × 10^7 ^in the right ear and EDIM 4 μg/ml in the left ear (Figure 4). As a control, the same rotaviruses were also administered to non-inoculated mice (n = 15) of the same age. There was little measurable swelling compared to the control with RRV (data not shown). It has been shown previously that a rotavirus-specific DTH response was not elicited when neonatal mice were infected at any time point after primary infection [5]. EDIM induced a small but significant (p = 0.01) increase in ear swelling in the EDIM group (group B) compared to the control, indicating the induction of specific cellular immunity. The RRV (group A), RRV+EDIM (group C) and Gastrogard-R^® ^(group D) groups were all comparable to the control. This might indicate that mice who received a primary infection at a young age show a DTH suppression when re-infected at an older age.

### *In vitro *Concanavalin A and rotavirus-specific proliferation in spleen cells

**Figure 5:**
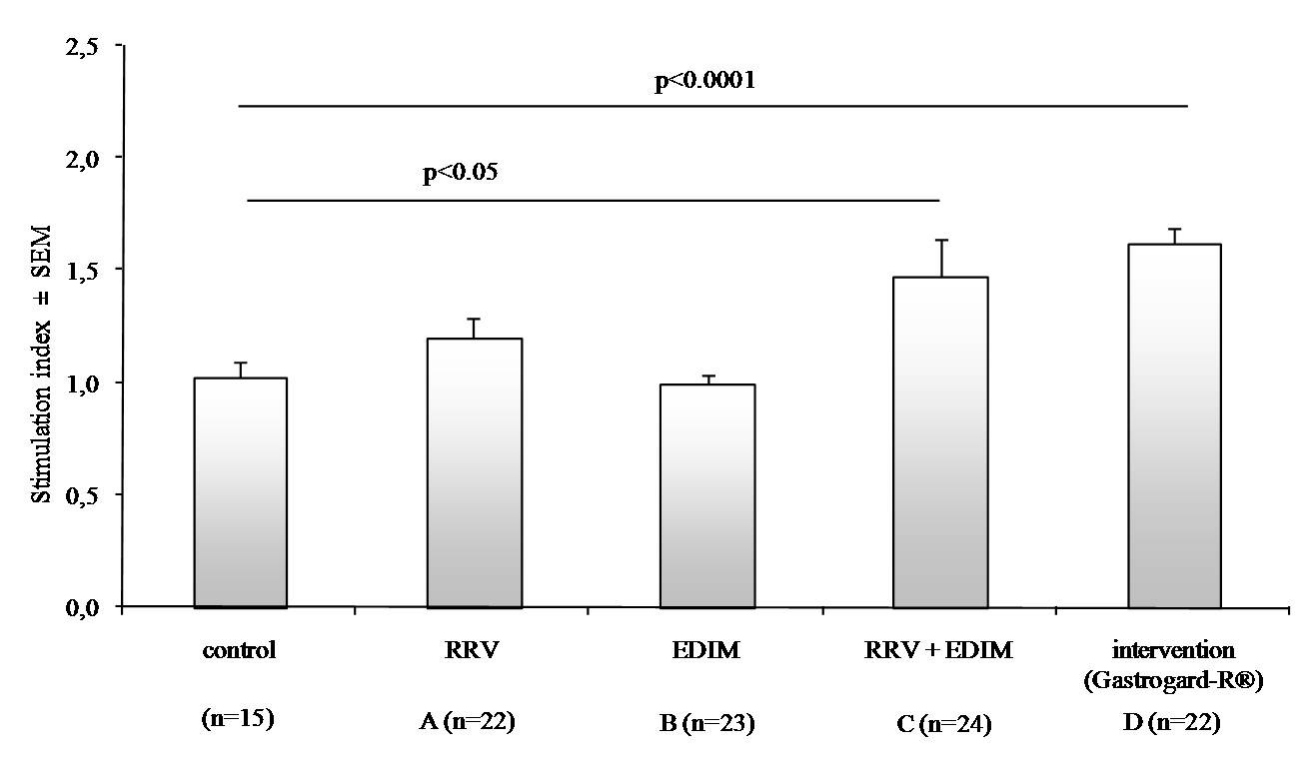
**T-cell proliferation by antigen-specific stimulation with rotavirus RRV**. Spleen cells of individual mice were isolated at day 28 and 1 × 10^6 ^cells were stimulated with 5 × 10^7 ^CCID_50 _UV-inactivated RRV for 5 days. Proliferation of T cells was determined by measuring incorporation of tritium-thymidine. Bars represents the stimulation index (RRV stimulated cells/non-stimulated cells) ± SEM per group. The RRV + EDIM group as well as the Gastrogard-R^® ^group showed a significant increase in T cell proliferation (p < 0.05 and p < 0.0001 respectively) compared to non-infected mice (control).

To analyze T cell responses, spleen cells from all study groups were isolated and *ex vivo *restimulated with either Concanavalin A (Con A), RRV or EDIM. As a control, spleen cells of non-inoculated mice (n = 15) of the same age were used. Con A induced a significant stimulation of T cell proliferation with an average stimulation index (Con A stimulated cells/non-stimulated cells) of approximately 30. However, there were no differences seen between the groups (data not shown). Incubation of the spleen cells with EDIM showed no proliferation (data not shown), but RRV incubation induced proliferation of T cells as shown in Figure 5. The RRV+EDIM group (group C) as well as the Gastrogard-R^® ^group (group D) showed a significant increase in T cell proliferation (p < 0.05 and p < 0.0001 respectively) compared to non-infected mice. These results suggest that multiple infections are needed to acquire a sufficient amount of rotavirus-specific memory T cells in the spleen to be able to re-stimulate these T cells *in vitro*.

### Rotavirus-specific serum IgM and IgG antibodies

**Figure 6:**
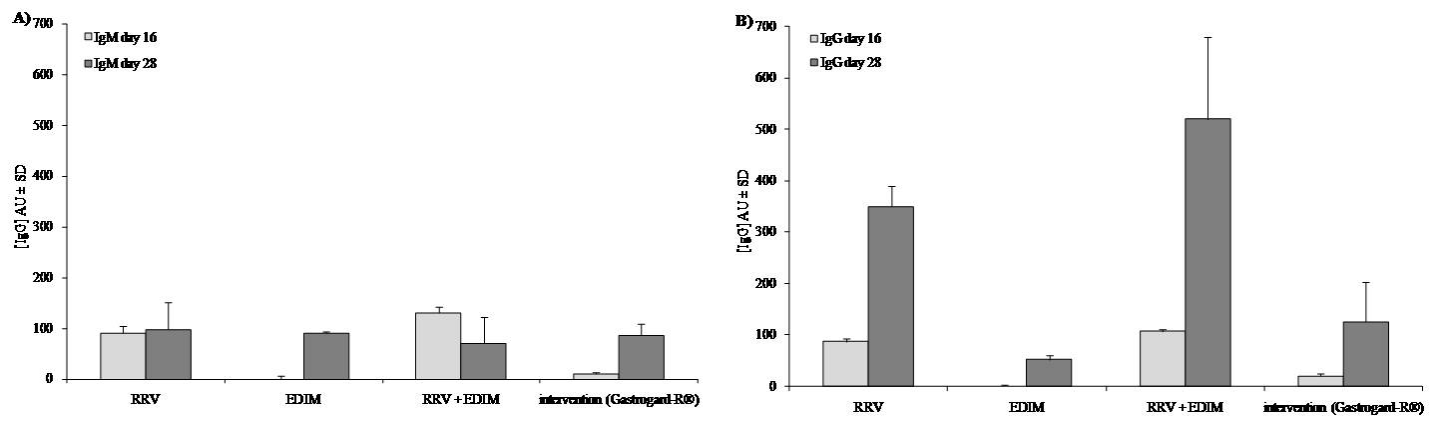
**Rotavirus-specific IgM and IgG antibody titers in serum**. Rotavirus-specific IgM (A) and IgG (B) antibody titers in arbitrary units (AU) ± SD in serum collected at day 16 (pre-EDIM serum) and at day 28 (post-EDIM serum). The serum at day 16 is a pooled serum collected from all animals within a group, at day 28 the sera were collected and tested individually (RRV n = 22; EDIM n = 23; RRV+EDIM n = 24; intervention Gastrogard-R^® ^n = 22) and the mean titers are depicted.

Rotavirus-specific IgM and IgG were measured in the serum individually collected at day 16 and then pooled per group (pre-EDIM serum) and in the individual sera of the pups collected at day 28 (post-EDIM serum). RRV inoculation at day 7 resulted in rotavirus-specific IgM titers (Figure 6A) in the pre-serum of about 100 AU in the RRV (group A) and RRV+EDIM (group C) groups. There was no rotavirus-specific IgM detectable in the EDIM group (group B). Surprisingly, even though clinical symptoms during the RRV infection were inhibited by Gastrogard-R^® ^(group D), a low rotavirus-specific IgM level (10 AU) was measured in this group. In the post EDIM inoculation sera, the rotavirus-specific IgM titers of the RRV and RRV+EDIM groups were not different from the pre-serum, but the rotavirus-specific IgM antibody titer was increased in the EDIM group as well as the Gastrogard-R^® ^group to 90 AU.

The rotavirus-specific IgG titers (Figure 6B) in the pre-serum showed similar results as the rotavirus-specific IgM with titers of about 100 AU in the RRV and RRV+EDIM groups, non-detectable in the EDIM group and low but detectable (20 AU) in the Gastrogard-R^® ^group. Inoculation with EDIM increased the titers of rotavirus-specific IgG in all groups (RRV 350 AU; RRV+EDIM 520 AU; EDIM 50 AU; Gastrogard-R^® ^125 AU).

### Rotavirus-specific serum IgG subclass antibodies

**Figure 7:**
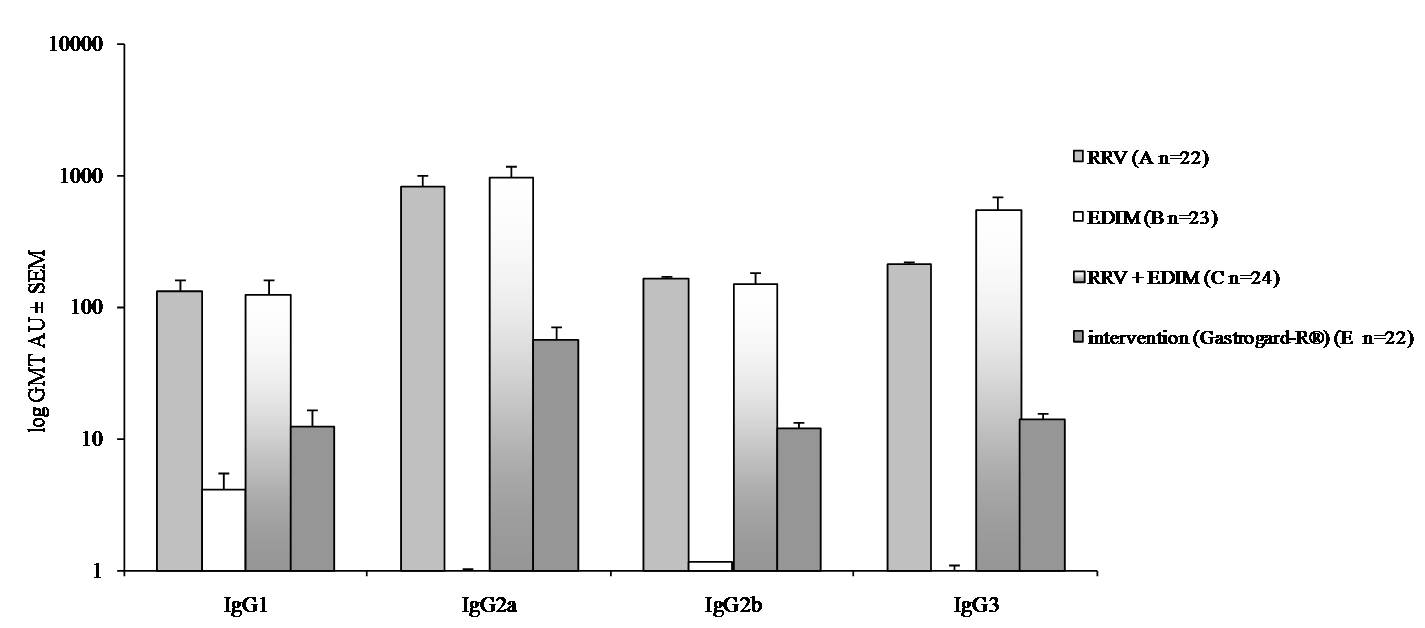
**Rotavirus-specific IgG-subclass antibodies in serum**. Geometric mean titers (GMT) in arbitrary units (AU) ± SEM of the rotavirus-specific IgG-subclass antibodies IgG1, IgG2a, IgG2b and IgG3 in serum individually collected at day 28, depicted per antibody.

The rotavirus-specific IgG subclasses IgG1, IgG2a, IgG2b and IgG3 were measured in the pool serum, individually collected and then pooled per group, at day 16 (pre-EDIM serum) and in the individual sera of the pups collected at day 28 (post-EDIM serum). Like rotavirus-specific IgG titers, the pre-serum showed detectable levels of all subclasses in the RRV and RRV+EDIM groups, 2 times lower in the Gastrogard-R^® ^group and non-detectable in the EDIM group (data not shown). The rotavirus-specific IgG subclass titers in the post-serum were markedly higher and in Figure 7 the geometric mean titers (GMT) per group are shown. Primary rotavirus inoculation at day 7 of age (RRV; group A) showed the following antibody titers; IgG2a antibodies (GMT 838 AU), IgG3 (GMT 213 AU), IgG2b (GMT 164 AU) and IgG1 (GMT 131 AU). A secondary EDIM inoculation at day 17 of age (RRV+EDIM; group C) showed similar IgG2a (GMT 981 AU), IG2b (GMT 151 AU) and IgG1 (GMT 125 AU) levels, only the amount of IgG3 seemed to be elevated (GMT 555 AU) but this increase was not significant. Primary inoculation with EDIM at day 17 of age (group B) resulted in low antibody levels for all subclasses (range 1-4 AU). Antibody levels in the mice receiving Gastrogard-R^® ^were significantly lower than the RRV+EDIM group (IgG1 p = 0.035, IgG2a p = 0.024, IgG2b p = 0.025, IgG3 p = 0.001). However, this Gastrogard-R^® ^group showed significantly higher levels of antibodies than the EDIM group (IgG1 p = 0.028, IgG2a p = 0.002, IgG2b p = 0.002, IgG3 p = 0.007). These results indicate that although Gastrogard-R^® ^had completely inhibited rotavirus-induced diarrhea during a primary infection, some infection had occurred or stimulation of the immune system because B cells were activated and rotavirus-specific IgG (subclass) antibodies were produced.

## Discussion

In this study a neonatal mouse model, originally developed by VanCott et al. [12], was modified to investigate the effect of nutritional intervention (Gastrogard-R^®^) during a primary (heterologous) rotavirus infection and/or on a secondary (homologous) rotavirus infection. Gastrogard-R^® ^is prepared from the colostrum of hyperimmunised cows and contains high antibody titers against four human rotavirus serotypes. In parallel, the neonatal mouse model could provide better insight into the immunological response to rotavirus since mechanism of rotavirus protection and rotavirus clearance in mice are still not fully understood. The ability of a neonatal mouse or human to generate sufficient immune effectors needed for protection after gastrointestinal virus infection is dependent on its state of immunological maturity. Specific immune cell functions as well as the gastrointestinal tract mature in neonatal mice through the weaning period, while the numbers of immune cells in inductive and effector sites increase gradually [14]. Many studies have been performed to clarify the immune response to rotavirus infection. Clearance of rotavirus can occur T cell independent [15,16] as well as B cell/antibody independent [17,18]. As for protection to rotavirus reinfection, B cells are absolutely necessary for long-term protection against rotavirus re-infection [7]. On the other hand, T cells are important for antiviral immunity in mice as well. CD4^+ ^T cells are essential for the development of more than 90% of the rotavirus-specific intestinal IgA and their presence seems to be critical for the establishment of protective long term memory responses [15]. Moreover, murine rotavirus-specific CD8^+ ^T cells can mediate short-term partial protection against reinfection [19]. These data implicates that there is not one specific route that leads to rotavirus clearance and/or protection to rotavirus re-infection, but that both B cell as well as T cell-dependent and independent mechanisms can lead to clearance of infection and long-term maintenance of protection [20]. Within the model as described herein, both aspects of immune responses related towards protection can be studied.

Sheridan et al. was one of the first to describe a mouse model studying rotavirus-specific immunity [5]. Mice (CD-1) were infected orally with EDIM virus at 1, 7 or 21 days of age. Severe disease was observed in animals infected at 1 day of age and lasted for at least 9 days. Disease was observed in mice infected at day 7 of age also, but was less severe and lasted only 5 days. Mice infected at 21 days of age did not show any evidence of clinical illness. These findings were comparable to our study where the mice were inoculated with RRV at day 7 of age and illness and diarrhea was seen for 5 days in approximately 70% of the animals. If the animals were supplemented with rotavirus-specific antibodies (Gastrogard-R^®^) orally, the animals were protected completely from rotavirus-induced diarrhea. Inoculation of EDIM at day 17 of age did not result in any clinical symptoms and infection was measured by the analysis of rotavirus shedding in feces. Fecal viral shedding after a secondary EDIM inoculation showed that a primary rotavirus infection protected against viral shedding by 81% during a secondary inoculation. Administration of Gastrogard-R^®^, which completely protected the mice from diarrhea and illness during a primary infection, showed no protection during the secondary inoculation though the viral shedding seemed to disappear more rapidly compared to the group which received only EDIM without primary RRV inoculation.

Delayed-type hypersensitivity (DTH) is an important *in vivo *manifestation of cell-mediated immune responses. In our study, a rotavirus-specific DTH using EDIM was elicited at day 27 of age. In mice only inoculated with RRV at day 7, no DTH response was measurable compared to the control group. The mice receiving only EDIM at the age of 17 days however showed a significant DTH response to EDIM. This DTH disappeared in the mice which have been inoculated with both RRV and EDIM. Thus, not only adult mice that were re-infected after a primary infection showed a suppressed DTH as seen previously by Sheridan et al [5] but also mice who received the primary infection at a young age and a re-infection at an older age showed the same DTH suppression. Cellular responses to rotavirus were also analyzed by *ex vivo *restimulation of T cells isolated from the spleen with UV-inactivated rotavirus. Inactivation by exposure to UV radiation destroys the integrity of rotavirus RNA and also removes the non-specific stimulatory effects of the virus when assayed on non-immune cells. A disadvantage of UV-inactivation over live virus is that inactivated virus has been shown to produce a lower level of proliferation than that induced by live virus [21]. In the present experiments, the proliferation level was low, although a significant increase in T cell proliferation was seen in the mice receiving both RRV and EDIM and also in the Gastrogard-R^® ^group. In these mice, even though during a primary infection clinical illness was completely blocked, the immune system was activated.

Much controversy still exists as to whether serum antibodies against rotavirus are directly involved in protection or merely reflect recent infection, leaving the protective role to mucosal or cell-mediated immunity. Reviewed data from a variety of studies in humans suggest that serum antibodies, if present at critical levels, are either protective themselves or are an important and powerful correlate of protection against rotavirus disease [22]. Previous studies in infant mice, rabbits and humans have determined that rotavirus-specific IgM levels increase during the acute-phase of infection (before 7 days p.i.) and then decrease gradually. Therefore, rotavirus-specific IgM is seen as a marker of primary infection. Rotavirus-specific IgA and IgG levels were increased in the convalescent-phase of the infection [5,23,24]. Administration of RRV at day 7 resulted in the development of an antibody titer after 21 days, predominantly of the IgG2a subclass. Similar subclass restriction after virus infections was seen previously [25,26]. Administration of EDIM to previously RRV inoculated mice did not result in an increase of the antibody titer. Administration of EDIM at day 17 without a previous RRV inoculation resulted in normal levels of rotavirus-specific IgM and a small amount of rotavirus-specific IgG (subclass) antibodies, most likely due to the fact that 10 days p.i. is too early to measure the development of IgG antibodies. Intervention with Gastrogard-R^® ^showed low antibody titers, but still significantly higher than the group who were inoculated with EDIM only, indicating that the rotavirus, although not able to induce diarrhea during the primary infection, could still provoke an antibody reaction.

Gastrogard-R^® ^is prepared from colostrum of hyperimmunised cows and contains high antibody titers against four human rotavirus serotypes, as measured in a virus neutralisation test [13]. The efficacy of passive immunization was established in calves which were immunized by subcutaneous injection of colostral whey with a high IgGl rotavirus antibody titer and challenged with virulent bovine rotavirus 48 h later. Calves were protected from rotavirus infection and diarrhea. Results further indicated that circulating IgG1 antibody appeared in the gastrointestinal tract of neonatal calves [27]. Previous research already showed that rotavirus antibody activity survived passage through the human gastrointestinal tract [28]. Gastrogard-R^® ^is used as prophylactic treatment of 'at risk' children aged one month to three years to prevent diarrhea due to rotavirus infection. The efficacy of treatment with Gastrogard-R^® ^was established in a clinical trial in children aged 3 to 15 months [13]. The *in vitro *inhibitory effect of Gastrogard-R^® ^was established in our laboratory in a rotavirus (RRV) titration assay using MA-104 cells (data not shown). In this assay Gastrogard-R^® ^was shown to have a strong inhibitory effect on the infectivity of rotavirus with an IC_50 _of 1 μg/ml. Bovine milk and bovine milk constituents like lactadherin have been studied on their inhibitory activity *in vitro *and in *in vivo *rotavirus models [29]. Various compounds present in whey protein concentrate can ameliorate the severity and incidence of experimental rotaviral diarrhea and modulate the mucosal and systemic immune in suckling rats [30] and suckling mice [31]. However, none of these dairy compounds were able to completely inhibit clinical symptoms during a primary rotavirus infection, which is usually attended with the most severe clinical symptoms like diarrhea and vomiting [32].

## Conclusions

In this study was found that oral administration of rotavirus antibodies can completely protect neonatal mice from clinical symptoms of illness during a primary rotavirus infection. Furthermore, an enhanced rotavirus-specific T cell proliferation and a small but detectable level of rotavirus-specific antibodies were found after re-infection suggesting improved T cell responses and a slight B cell response. Also shedding of rotavirus after EDIM inoculation seemed to disappear more rapidly in mice treated with rotavirus antibodies than in mice inoculated with EDIM alone. These results indicate that even though symptoms of a primary rotavirus infection were prevented, an activation of the immune system was still detectable. Preventing a primary infection by using Gastrogard-R^®^, activation of the immune system can still occur and could be helpful during re-infection knowing that both arms of the immune system play a pivotal role in immunity to rotavirus infection. These data show that this intervention model can be used for studying clinical symptoms as well as immune responses required for protection against viral re-infection.

## Abbreviations

CCID_50_: cell culture infective dose; Con A: Concanavalin A; DTH: delayed-type hypersensitivity; EDIM: epizootic-diarrhea infant-mouse; RRV: rhesus rotavirus; SA-11: simian rotavirus

## Competing interests

The authors declare that they have no competing interests.

## Authors' contributions

KK participated in the design and coordination of the *in vivo *study, participated in the *in vitro *experiments and drafted the manuscript. MM participated in the design *in vivo *study and helped to draft the manuscript. AC participated in execution and analysis of the *in vitro *experiments. GvA participated in the design of the *in vivo *study and was responsible for the execution of the *in vivo *study. JG has been involved in drafting the manuscript for important intellectual content and has given final approval for the manuscript to be submitted. BvtL participated in the design of the *in vivo *study and helped to draft the manuscript. All authors read and approved the final manuscript.
